# *Antrodia cinnamomea* boosts the anti-tumor activity of sorafenib in xenograft models of human hepatocellular carcinoma

**DOI:** 10.1038/s41598-018-31209-8

**Published:** 2018-08-27

**Authors:** Wei-De Wu, Pin-Shern Chen, Hany A. Omar, El-Shaimaa A. Arafa, Hung-Wei Pan, Jingyueh Jeng, Jui-Hsiang Hung

**Affiliations:** 1 Department of Biotechnology, Chia Nan University of Pharmacy and Science, Tainan, Taiwan; 2 Sharjah Institute for Medical Research and College of Pharmacy, University of Sharjah, Sharjah, UAE; 3 Department of Clinical Sciences, College of Pharmacy, Ajman University, Ajman, UAE; 4 Department of Pharmacology, Faculty of Pharmacy, Beni-Suef University, Beni-Suef, Egypt; 5 Department of Medical Education and Research, Kaohsiung Veterans General Hospital, Kaohsiung, Taiwan; 6 Drug Discovery and Development Center, Chia Nan University of Pharmacy and Science, Tainan, Taiwan

## Abstract

Hepatocellular carcinoma (HCC) has been recognized worldwide as one of the major causes of cancer death. The medicinal fungus *Antrodia cinnamomea* (*A*. *cinnamomea*) has been served as a functional food for liver protection. The aim of the present study was to investigate the potential activity of *A*. *cinnamomea* extracts as a safe booster for the anticancer activity of sorafenib, a multi-kinase inhibitor approved for the treatment of HCC. The biologically active triterpenoids in the ethanolic extracts of *A*. *cinnamomea* (EAC) were initially identified by HPLC/LC/MS then the different extracts and sorafenib were assessed *in vitro* and *in vivo*. EAC could effectively sensitize HCC cells to low doses of sorafenib, which was perceived via the ability of the combination to repress cell viability and to induce cell cycle arrest and apoptosis in HCC cells. The ability of EAC to enhance sorafenib activity was mediated through targeting mitogen-activated protein (MAP) kinases, modulating cyclin proteins expression and inhibiting cancer cell invasion. Moreover, the proposed combination significantly suppressed ectopic tumor growth in mice with high safety margins compared to single-agent treatment. Thus, this study highlights the advantage of combining EAC with sorafenib as a potential adjuvant therapeutic strategy against HCC.

## Introduction

Hepatocellular carcinoma (HCC) affects approximately one million people worldwide annually^1–3^. Common causes of death among HCC patients include the emergence of new primary HCC tumors and metastasis are the most^4^. The available curative measures in HCC include surgical removal, percutaneous tissue destruction or liver transplantation, which are only applicable in 10–20% of patients due to poor liver function^5–7^. RAF/MEK/ERK pathway, triggered by HCV, HBV infection or mitogens, as well as other cancer cell signaling cascades, play an important role in liver tumorigenesis and poor prognosis^8–10^.

Sorafenib (Nexavar™), is one of the targeted therapies known as kinase inhibitors already approved for HCC treatment^11^. Sorafenib targets mainly RAF signaling in addition to VEGF (vascular endothelial growth factor), PDGF (platelet derived growth factor), and c-Kit, which results in significant antiproliferative and antiangiogenic activity in HCC^12–14^. To expand the efficacy of sorafenib, many studies have demonstrated a successful therapeutic combination of sorafenib and other agents targeting parallel signaling pathways. For example, the combination of sorafenib with OSU-2S synergistically enhanced the antiproliferative effects on HCC cells, and PKCδ and p53 were involved in regulation of sorafenib/OSU-2S-induced cell death^15^. Many studies have indicated the synergy of sorafenib with different bioactive components in plant extracts such as corosolic acid in *Actinidia chinensis*^16^, resveratrol in grapes, peanuts and red wine^17^ and wogonin (5,7-dihydroxy-8-methoxyflavone) in *Scutellaria baicalensis*^18^.

*Antrodia cinnamomea* (*A*. *cinnamomea*) is a unique edible medicinal fungus originating in Taiwan, that has been used in the management of food intoxication, cancer prevention and to improve liver functions^19–22^. Cytotoxic, anti-inflammatory and hepatoprotective properties of the active components of *A*. *cinnamomea* extracts have been reported previously^23–25^. In addition, camphorataimide B, an active component of *A*. *cinnamomea*, exhibited potent cytotoxic activities in lung cancer cells (A549), leukemia cells (HL-60) and breast cancer cells (MCF-7) and (MDA-MB-231)^26^. Furthermore, 4-acetylantroquinonol B isolated from *A*. *cinnamomea* inhibited cell proliferation, mTOR phosphorylation and VEGF production in HCC cells^27^. However, the introduction of *A*. *cinnamomea* extract as an adjuvant medication to sorafenib in HCC therapeutic protocols has not been explored previously.

Therefore, our aim was to explore the cytotoxic activity of sorafenib and *A*. *cinnamomea* extract against human liver cancer cells with a special emphasis on the possible synergistic mechanisms via ERK signaling pathways, both *in vitro* and *in vivo*. The outcome of the present study may provide a basis to develop a novel formula of EAC extract to be used in HCC therapeutic protocols.

## Results

### Identification of biologically active triterpenoids in EAC extracts

**Figure 1:**
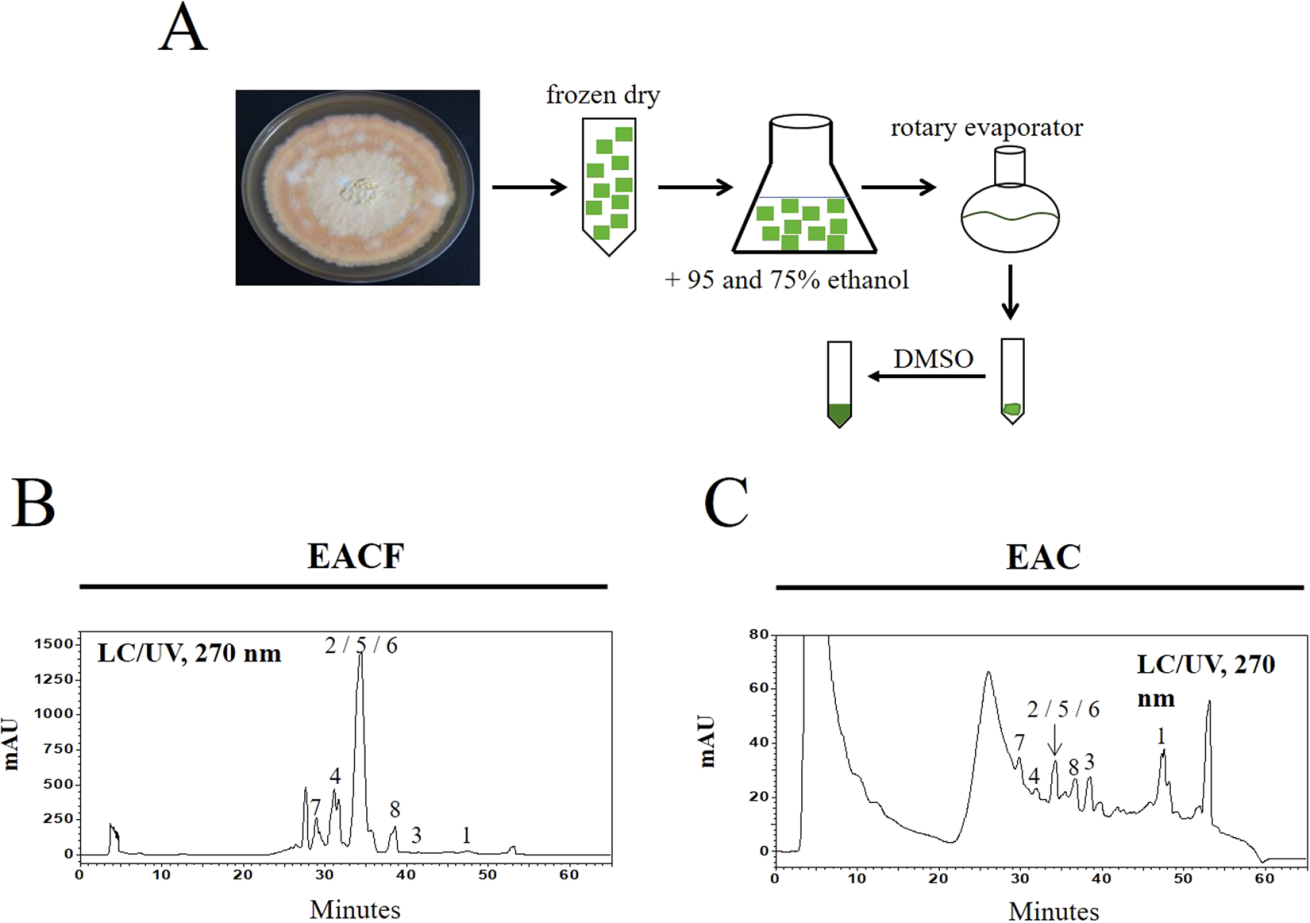
The preparation and identification of the major triterpenoids in *A*. *cinnamomea* extract. (**A**) A flowchart showing the extraction protocol of *A*. *cinnamomea* cultivated on agar plates. The dried agar plates were extracted with ethanol and concentrated by a rotary evaporator. (**B**, **C**) The dried extracts of EACF or EAC were dissolved in DMSO, and 10 mg/ml of total extracts were analyzed by HPLC/LC/MS.

For *A*. *cinnamomea* cultivation, *A*. *cinnamomea* strain was seeded in M25 culture medium and incubated at 25 °C for 50 days. The frozen, dried plates were then extracted with 95% and 75% ethanol every 3 days. The total crude extracts were concentrated using a rotary evaporator (Fig. 1A). To identify the metabolite profile of the samples obtained from different growth substrates, the HPLC fingerprint of the wild fruiting body ethanolic extract of *A*. *cinnamomea* (EACF) was used as a standard (Fig. 1B). To evaluate the bioactive compounds in EAC, 10 mg/ml EAC was determined by HPLC/LC/MS with UV (Fig. 1C). Many compounds have been identified and listed in Table 1. The index compounds were: (1) methyl antcinate B, (2) methyl antcinate A, (3) dehydroeburicoic acid, (4) antcin A, (5) antcin B, (6) antcin K, (7) 15α-acetyl dehydrosulphurenic acid, (8) dehydrosulphurenic acid, (9) 3β,15α-dihydroxy-lanosta-7,9(11),24-triene-21-oic acid, (10) zhankuic acid C. Previous studies have demonstrated that those major triterpenoids in *A*. *cinnamomea* play an important role in its anticancer activity^28^. Our result indicated that EAC extract contains those important triterpenoids as detected by UV, total ion chromatogram (TIC) and LC/MS/MS analysis (Supplementary Material Part 1).

**Table 1:** The major triterpenoids in *A*. *cinnamomea*.

No.	M.W.	Compound
1	482	Methyl antcinate B
2	468	Dehydroeburicoic acid/Antcin B/Methyl antcinate A
3	526	15α-acetyl dehydrosulphurenic acid
4	470	3β,15α-dihydroxy-lanosta-7,9(11),24-triene-21-oic acid
5	484	Dehydrosulphurenic acid
6	486	Zhankuic acid C
7	488	Antcin K
8	454	Antcin A

**M**.**W**., Molecular weight (g/mol).

### Inhibition of HCC cell viability by sorafenib and EAC

**Figure 2:**
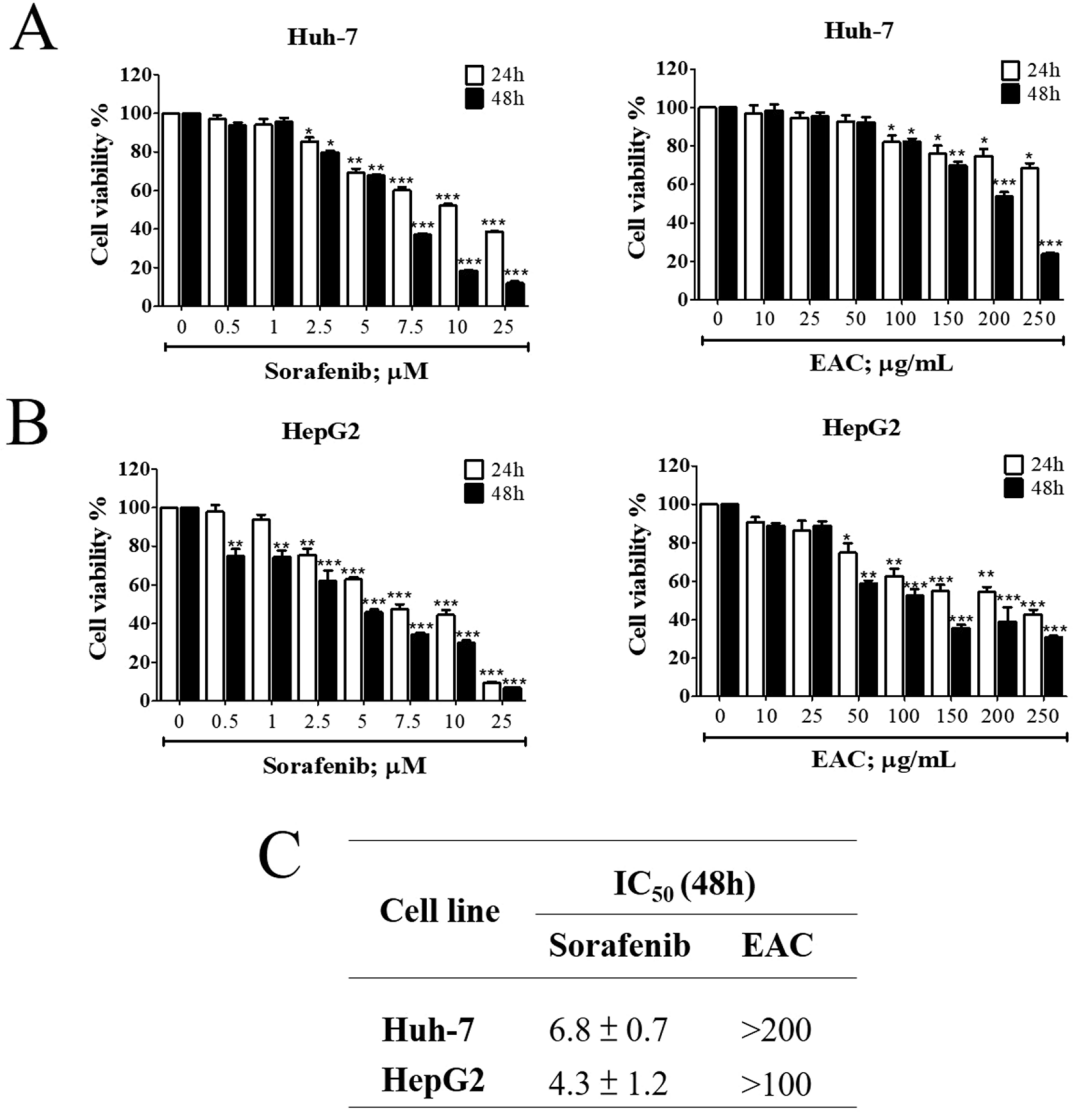
Antiproliferative effects of sorafenib and EAC in HCC cell lines. (**A**) Huh-7 cells and (**B**) HepG2 cells were exposed to sorafenib and EAC at the indicated concentrations in 10% FBS-Supplemented DMEM for 24 and 48 h, and cell viability was assessed by MTT assays. Columns, mean; bars, SD (*n* = 6). *P < 0.05; **P < 0.01; ***P < 0.001. (**C**) The IC_50_ values of sorafenib and EAC in two HCC cell lines after treatment for 48 h and cell viability was measured by MTT assay.

MTT assay was used to assess the effect of sorafenib and EAC on liver cancer cell viability after 48 h treatment. Sorafenib reduced cell viability in Huh-7 and HepG2 cells in a dose-dependent manner, the same was observed in EAC treatment (Fig. 2A and B). HepG2 cells were more sensitive to both sorafenib and EAC compared to Huh-7 cells. The IC_50_ values of sorafenib in Huh-7 and HepG2 cells were 6.8 and 4.3 µM, respectively, while that of EAC were over 200 and 100 µg/ml, respectively (Fig. 2C).

### EAC sensitizes HCC cells to the cytotoxicity of sorafenib

**Figure 3:**
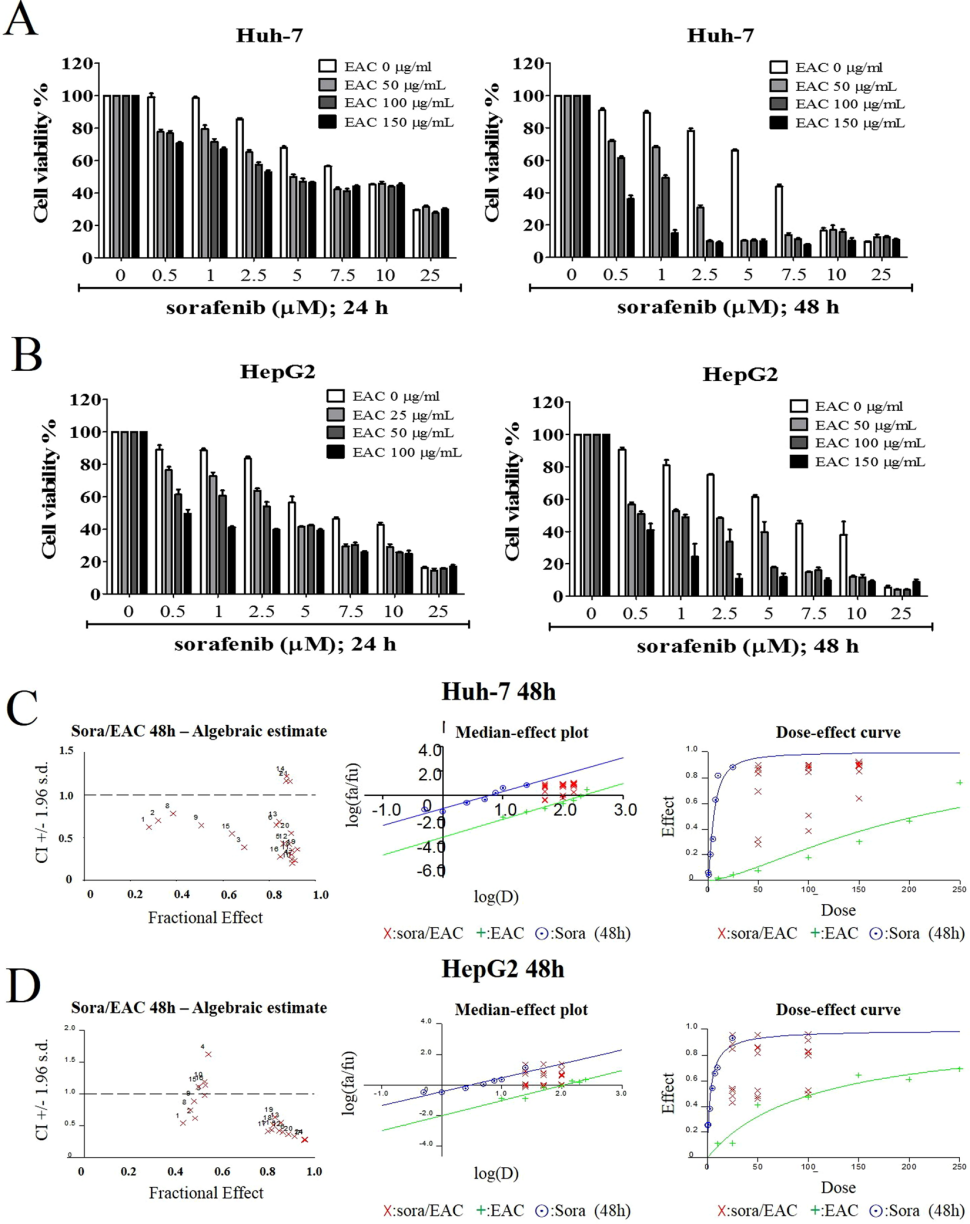
The synergistic combination between sorafenib and EAC on cancer cell viability. (**A**) Huh7 and (**B**) HepG2 cell lines were treated with sorafenib in the presence or absence of EAC at the indicated concentrations in 10% FBS-DMEM for 24 or 48 h, and cell viability was determined by MTT assays. Columns, mean; bars, SD (*n* = 6). (**C**,**D**) Sorafenib/EAC combination algebraic estimate, median effect plot and dose-effect curves calculated by Calcusyn software in the treated Huh7 and HepG2 cells.

To examine the ability of EAC to boost sorafenib’s anticancer activity, HCC cells were treated with different concentrations of sorafenib with or without EAC and the cell viability was assessed by MTT assay. The results indicated that EAC dose-dependently significantly sensitized Huh-7 and HepG2 cells to sorafenib-induced cell death (Fig. 3A, B). The combination index (CI) was analyzed using Calcusyn software version 2.1. as described previously^29^ (Fig. 3C, D). Different dose levels of sorafenib and EAC showed synergistic effects (Tables 2 and 3). In addition, Median-effect plot and Dose-effect curve were also determined (Fig. 3C, D). The results confirmed the synergy between sorafenib and EAC in Huh-7 and HepG2 cells.

**Table 2:** The fraction affected (Fa) and combination indices (CI) at the indicated combined doses of sorafenib and EAC in Huh-7 cells treated for 48 h.

Graph Points	Sorafenib (µM)	EAC (µg/ml)	Fa	CI
1	0.5	50	0.281679	0.625
2	1.0	50	0.320645	0.702
3	2.5	50	0.69312	0.386
4	5.0	50	0.898437	0.242
5	7.5	50	0.862123	0.428
6	10	50	0.831426	0.647
7	25	50	0.874458	1.169
8	0.5	100	0.385282	0.783
9	1.0	100	0.507049	0.646
10	2.5	100	0.901093	0.200
11	5.0	100	0.897358	0.299
12	7.5	100	0.88706	0.424
13	10	100	0.843152	0.687
14	25	100	0.876083	1.221
15	0.5	150	0.639206	0.550
16	1.0	150	0.849083	0.277
17	2.5	150	0.911273	0.234
18	5.0	150	0.899273	0.350
19	7.5	150	0.92258	0.362
20	10	150	0.895581	0.550
21	25	150	0.890966	1.160

**Table 3:** The fraction affected (Fa) and combination indices (CI) at the indicated combined doses of sorafenib and EAC in HepG2 cells treated for 48 h.

Graph Points	Sorafenib (µM)	EAC (µg/ml)	Fa	CI
1	0.5	50	0.429583	0.545
2	1.0	50	0.481666	0.621
3	2.5	50	0.523408	0.979
4	5.0	50	0.539493	1.626
5	7.5	50	0.847966	0.423
6	10	50	0.888559	0.372
7	25	50	0.958258	0.277
8	0.5	100	0.461752	0.749
9	1.0	100	0.477836	0.887
10	2.5	100	0.523025	1.194
11	5.0	100	0.81484	0.435
12	7.5	100	0.863284	0.405
13	10	100	0.856008	0.552
14	25	100	0.958449	0.285
15	0.5	150	0.498133	1.115
16	1.0	150	0.526855	1.141
17	2.5	150	0.798947	0.415
18	5.0	150	0.822307	0.510
19	7.5	150	0.829009	0.634
20	10	150	0.913834	0.336
21	25	150	0.96113	0.282

### Induction of cell cycle arrest and apoptosis by sorafenib/EAC treatment

**Figure 4:**
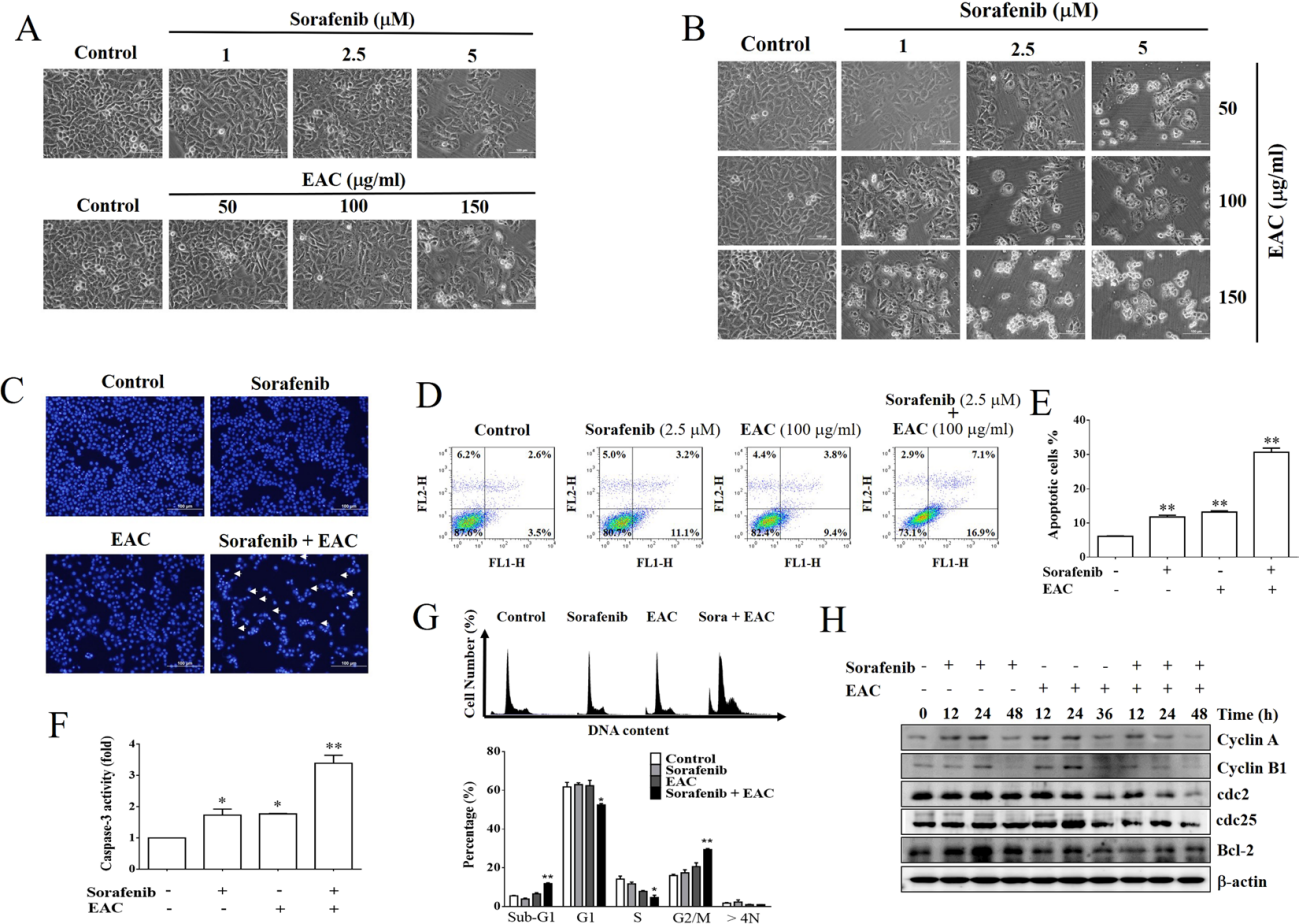
EAC sensitizes HCC cells to sorafenib-induced apoptosis in HCC cells. (**A**, **B**) Phase-contrast images showing the morphological changes of Huh-7 cells treated with sorafenib alone or in combination with EAC for 48 h. (**C**) Nuclear morphological changes of Huh-7 cells under a fluorescence microscope. The cells were treated for 48 h then stained with DAPI. (**D**) Annexin V-FITC/PI double staining analysis of apoptosis in Huh-7 cells treated with sorafenib (2.5 µM) and/or EAC (100 µM) for 48 h assessed by flow cytometry. (**E**) Results of Annexin V-FITC/PI double staining analysis. Columns, mean; bars, SD (*n* = 4). (**F**) Flow cytometry histograms of caspase-3 activity in Huh-7 cells treated with sorafenib (2.5 µM) and/or EAC (100 µM) for 48 h. Columns, mean; bars, SD (*n* = 4). (**G**) Cell cycle distribution using PI staining and flow cytometry of Huh-7 cells treated with sorafenib (2.5 µM) and/or EAC (100 µM) for 48 h. Columns, mean; bars, SD (*n* = 4). (**H**) Western blot analysis of Huh-7 cells lysate after the indicated treatments. *P < 0.05; **P < 0.01.

To assess the efficacy of sorafenib/EAC combination as a potential antitumor approach, the ability of the combination to elicit different aspects of apoptosis was tested. Huh-7 and HepG2 cells display different characteristics and genetic defects. A previous study indicated that the phosphorylation levels of ERK1/2 in Huh-7 cells were higher than in HepG2 cells^30^. Therefore, Huh-7 cells were used for further investigation. In the beginning, the effect of the combination on the cancer cell morphology was observed compared to single-agent treatment. It was obvious that the single treatment with sorafenib or EAC at the selected concentrations (Fig. 4A) was less active considering the induction of the characteristic progressive morphological changes of apoptosis, that were clearly observed in sorafenib/EAC combination (Fig. 4B). In addition, DAPI staining showed more apoptotic bodies (condensed chromatin), a characteristic of apoptosis, in sorafenib/EAC combination (Fig. 4C). Moreover, the induction of programmed cell death was detected by Annexin V staining assay (Fig. 4D). The percentage of apoptotic cells was significantly raised in Sorafenib/EAC combination by more than 20% compared to single-agent treatment (Fig. 4E). The induction of apoptosis was confirmed by a 3.5-fold rise in caspase-3 activity in sorafenib/EAC combination-treated Huh-7 cells compared to single-agent treatment (Fig. 4F).

Furthermore, the effects of sorafenib/EAC combination on cell-cycle distribution was assessed using flow cytometry. The cell cycle analysis in treated Huh-7 cells showed that sorafenib/EAC combination markedly increased the subG1 fraction of cells, from 5.5% (control) to 11.8% as an indication of cell death and DNA fragmentation (Fig. 4G). The combination also showed an arrest at G2/M phase, where the percentage of cells increased from 16% (control) to 29.4% accompanied by fewer cells in S phase (Fig. 4G). In addition, Western blot analysis showed the ability of sorafenib/EAC combination to moderately down-regulate different cyclins e.g. cyclin A, cyclin B1, cyclin D1, cdc2 and cdc25 and consequently regulating cell cycle progression (Fig. 4H).

### EAC boosted the inhibitory effect of sorafenib on ERK phosphorylation

**Figure 5:**
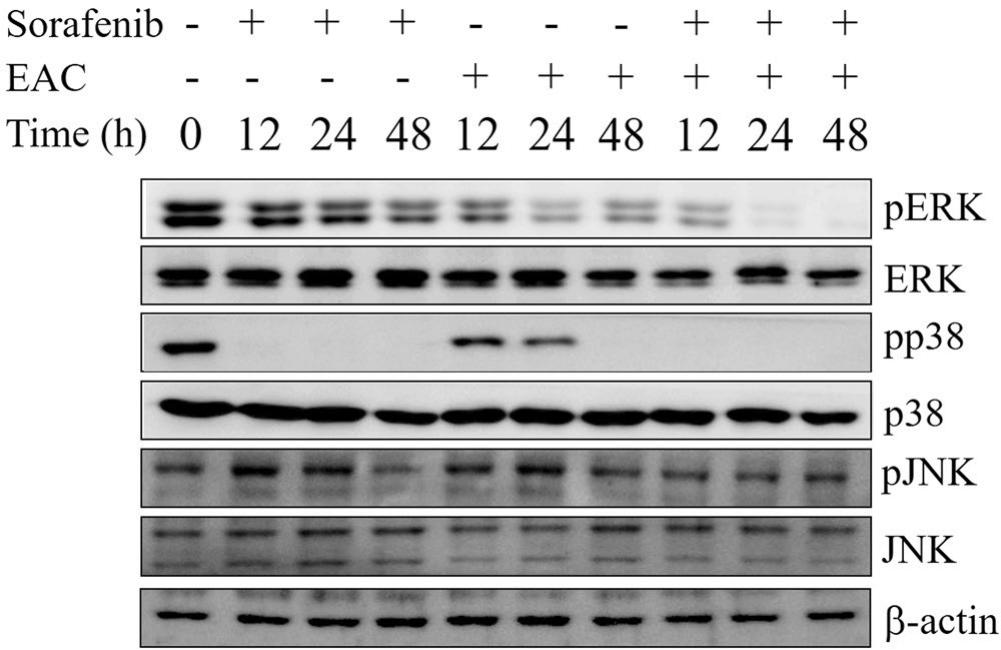
The effect of sorafenib/EAC combination on MAPKs. Western blots of Huh-7 cells treated with 2.5 µM sorafenib and/or 100 µg/ml EAC for 48 h. The total cell lysates were analyzed by Western blotting with antibodies against ERK, p-ERK, JNK, pJNK, p38, p-p38, and β-actin.

Since sorafenib mainly targets Ras/RAF/ERK pathway in HCC cells, the ability of EAC to modulate the mechanism of action of sorafenib was assessed using Western blot analysis. Results showed that sorafenib/EAC combination repressed ERK phosphorylation in Huh-7 cells (Fig. 5). The inhibition of pERK was more prominent in the combination treatment compared to single-agent treatment.

### Sorafenib/EAC combination Inhibits Huh-7 cells migration

**Figure 6:**
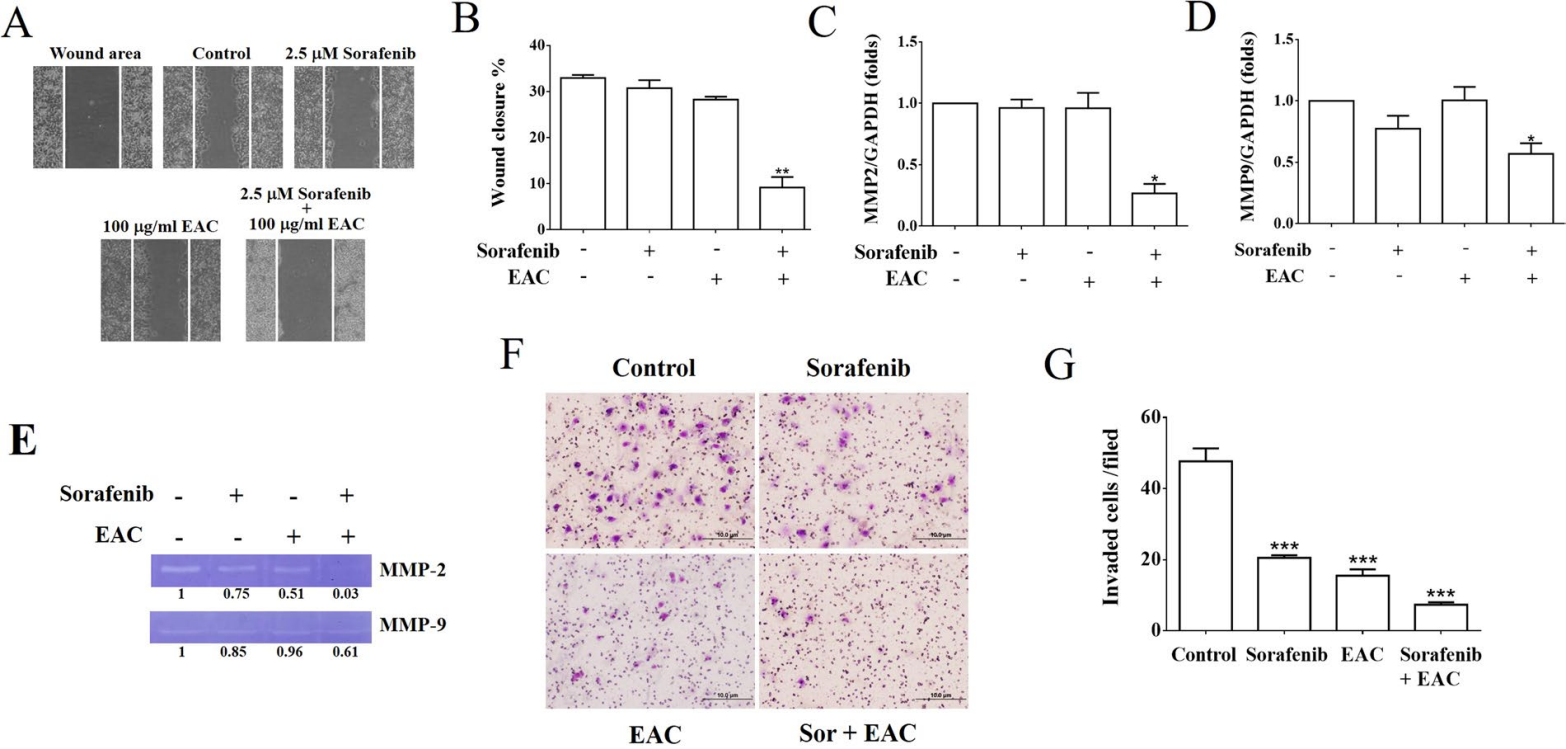
The inhibition of cancer cell migration and MMP expression by sorafenib/EAC combination. (**A**) Wound healing assay for Huh-7 cells exposed to 2.5 µM sorafenib and/or 100 µg/ml EAC for 48 h. The wounded region was observed by Olympus CK-2 inverted microscope (100× magnification). (**B**) The results of wound healing assay. Columns, mean; bars, SD (*n* = 4). (**C**, **D**) MMP-2 and MMP-9 expression in Huh-7 cells treated with 2.5 µM sorafenib and/or 100 µg/ml EAC for 48 h using Real-time PCR. Columns, mean; bars, SD (*n* = 4). (**E**) Zymography assay of Huh-7 cells treated with 2.5 µM sorafenib and/or 100 µg/ml EAC for 16 h in serum-free medium. The activity of MMP-2 and MMP-9 was determined by zymography assay with gelatin-containing SDS-PAGE. (**F**) Effect of sorafenib with or without EAC on Huh-7 cell invasion. Cells were treated with sorafenib and EAC at the indicated concentrations, and the invaded cells were photographed (200× magnification). (**G**) The invaded cells were counted in five random fields for each treatment. Data are presented as mean ± S.D (*n* = 5) of three independent experiments. *P < 0.05; **P < 0.01; ***P < 0.001 compared with the untreated control.

Cancer cell migration/metastasis is usually responsible for poor prognoses in cancer patients. The ability of sorafenib and EAC, as single-agents, to inhibit HCC cells migration and invasion through the suppression of matrix metalloproteinase (MMP) expression have been reported previously^31,32^. To test the possible synergistic effect of the combination on cell migration and MMPs expression, wound healing assay and real-time PCR were used. Results showed that sorafenib/EAC combination suppressed Huh-7 cells migration to the denuded zone, which was more significant than single-agent treatment (Fig. 6A, B). Corresponding inhibitions of mRNA expression levels of MMP-2 and MMP-9 were shown in Fig. 6C, D. Furthermore, the enzymatic activity of MMP-2/9 was determined by using zymography assay. Cells were treated with sorafenib, EAC and sorafenib/EAC in a serum-free culture medium. The result indicated that MMP-2 and MMP-9 enzyme activities were inhibited by sorafenib/EAC combination treatment (Fig. 6E). To determine the inhibitory effect of sorafenib with or without EAC treatment on the invasion of Huh-7 cells across the extracellular matrix, the invasion ability of Huh-7 during treatment was evaluated by Boyden chamber assay with the Matrigel-coated polycarbonate filter. The results indicated that sorafenib/EAC suppresses the invasion of Huh-7 cells across the Matrigel-coated filter. Treatment with sorafenib, EAC and sorafenib/EAC inhibited cell invasion by 57.0%, 67.5% and 86.4%, respectively (Fig. 6F, G).

### Sorafenib/EAC combination treatment is safe in mice

**Figure 7:**
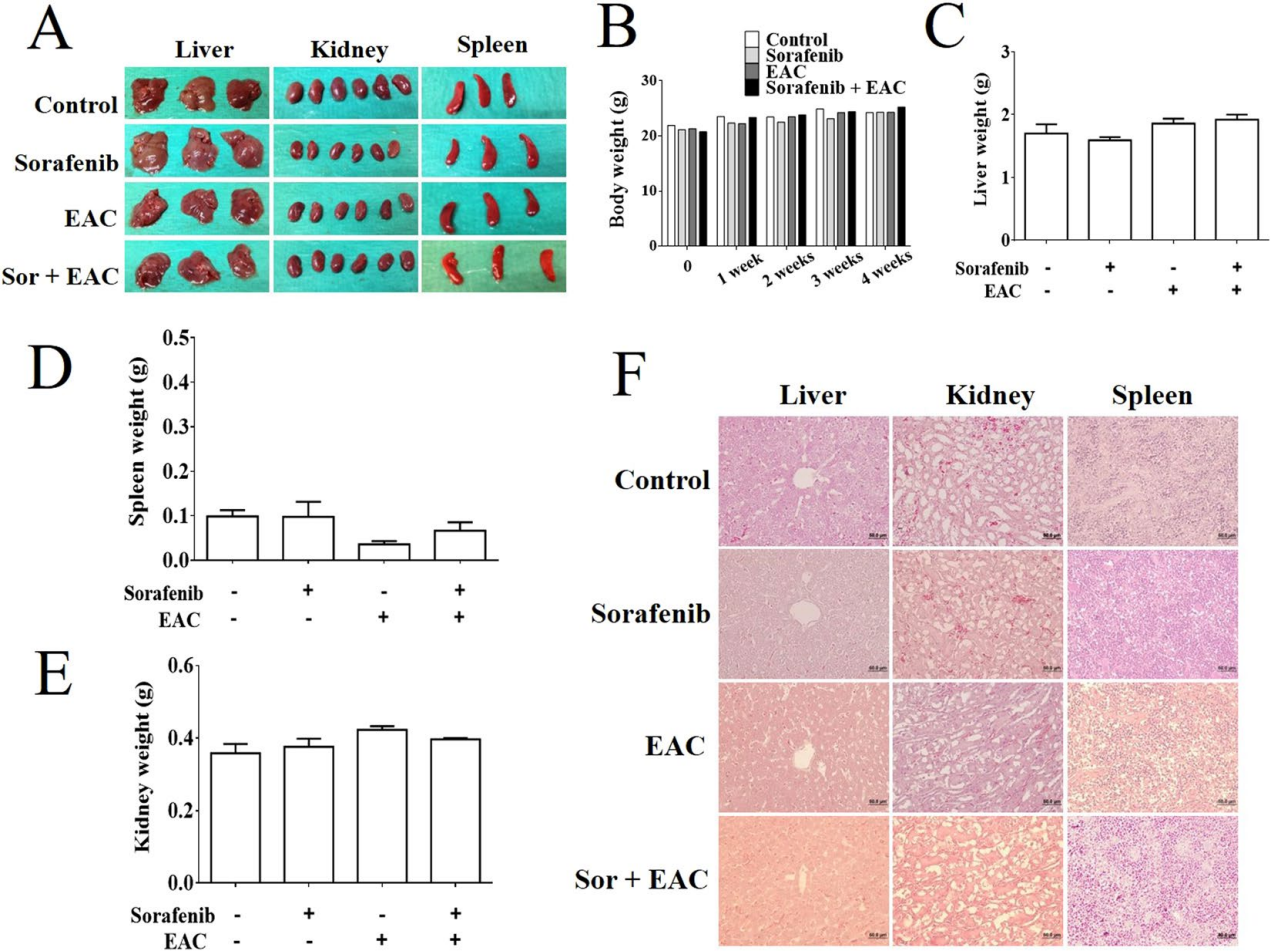
Assessment of sorafenib/EAC combination safety in mice. (**A**) The photographs of the livers, kidneys, and spleens of BALB/c mice treated with 2.5 mg/kg sorafenib and/or 100 mg/kg EAC (i.p.) every other day for 4 weeks. (**B**–**E**) The body, liver, spleen, and kidney weight changes through the treatment. Columns, mean; bars, SD (*n* = 3). (**F**) Representative images of the histopathological (H&E staining) analysis of liver, kidney, and spleen tissue sections (200× magnification) of the treated mice.

To analyze the safety of the proposed sorafenib/EAC combination treatment, BALB/c mice were treated with sorafenib/EAC combination or single-agents by i.p. injection every two days for 4 weeks. The animals were observed for any change in body weight during the study, then the animals were sacrificed, and the liver, kidney, and spleen were collected and analyzed for histopathological changes (Fig. 7A, F). The results showed that none of the single-agents or the combination treatments caused any significant changes in the animals’ body weight (Fig. 7B) nor in the liver, spleen or kidney weights (Fig. 7C–E). In addition, none of the used single-agents or combinations caused any significant histopathological changes nor obvious lesions in these organs (Fig. 7F).

### Sorafenib/EAC combination suppresses HCC xenograft growth *in vivo*

**Figure 8:**
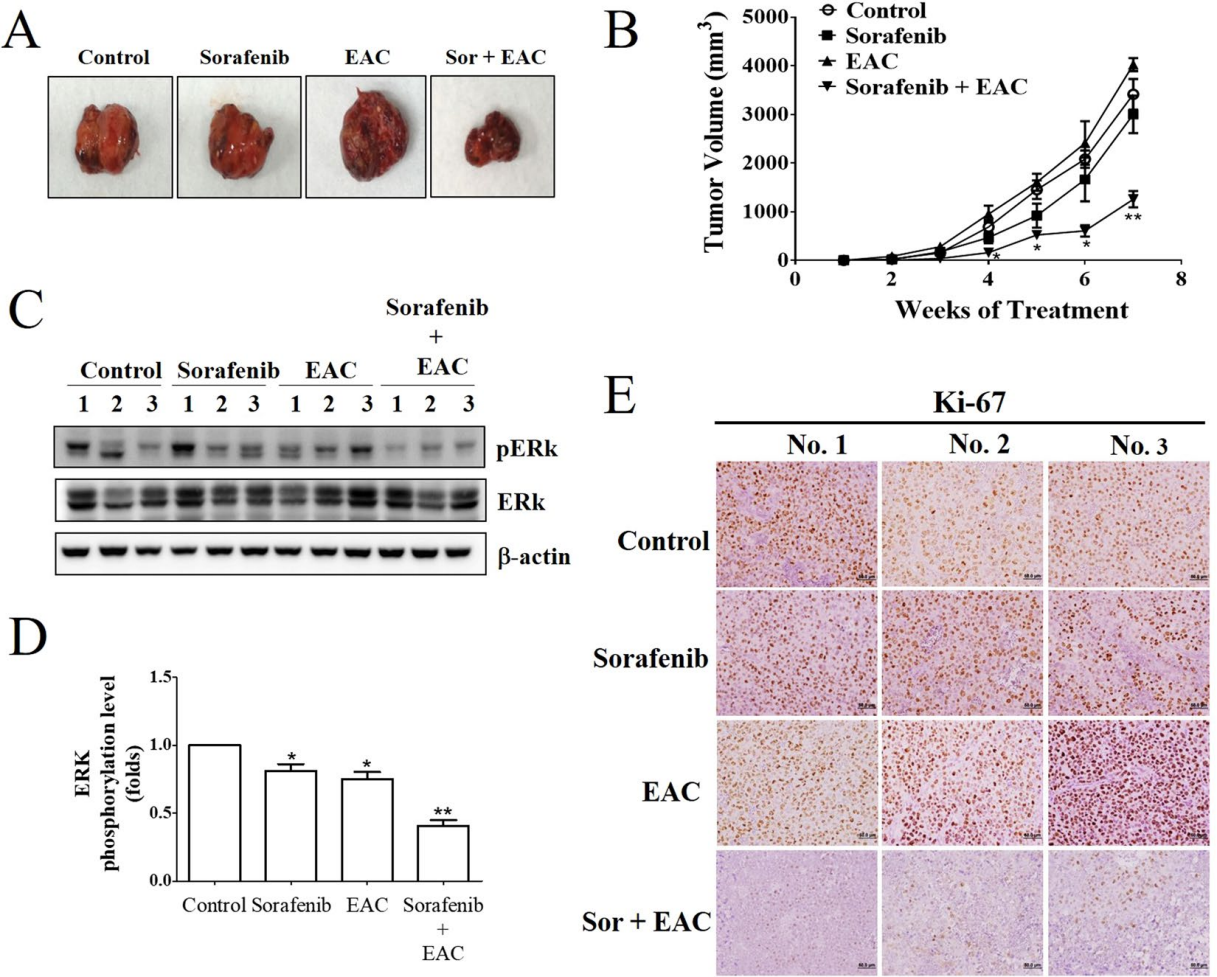
*In vivo* efficacy of sorafenib/EAC combination in an ectopic xenograft model of HCC. (**A**) Representative images showing Huh-7 xenograft tumors excised from NOD-SCID mice after the treatment with 2.5 mg/kg sorafenib and/or 100 mg/kg EAC (i.p.) every other day for 7 weeks. (**B**) Tumor volume changes over the 7 weeks of treatment. Points, mean; bars, SD. *P < 0.05; **P < 0.01. (**C**) Western blot analysis of ERK and p-ERK showing the drug activity in the homogenates of 3 representative subcutaneous Huh-7 tumors from each treatment group. (**D**) The phosphorylation levels of ERK in the tumor tissues. The Western blot images were quantified by using Image J software. Columns, mean; bars, SD (*n* = 3). *P < 0.05; **P < 0.01. (**E**) Immunohistochemistry images of Ki67 staining in representative xenograft tumors (200× magnification).

The treatment of NOD-SCID mice bearing established Huh-7 tumors with sorafenib, EAC or sorafenib/EAC combination for 7 weeks did not show any significant harmful signs nor decrease of mice body weight. The sorafenib/EAC combination caused a significant inhibition in the growth of ectopic Huh-7 tumors up to 52% compared to those treated with vehicle or single-agents (Fig. 8A, B). In addition, the ability of the combination to maintain the same mechanism of action *in vivo* was investigated by Western blot analysis of ERK phosphorylation status in tumor tissues. Results showed that sorafenib/EAC combination was able to inhibit ERK phosphorylation by 58% compared to vehicle-treated animals (Fig. 8C, D). Moreover, the mitotic index of the tumors was investigated via immunohistochemical analysis of Ki67 expression as a biomarker of a cell proliferation in the tumors section. Results showed that the expression of Ki67 in Huh-7 tumors was significantly reduced by sorafenib/EAC combination (Fig. 8E).

## Discussion

Poor prognosis and limited therapeutic efficacy have been usually associated with HCC. Resistance to most of the clinically-available anticancer agents posed the main barrier in the systemic treatment of HCC. Therefore, there is an urge for novel compounds or therapeutic approaches for HCC. Sorafenib is one of the targeted drugs approved for the treatment of advanced HCC^11^. Sorafenib is a kinase inhibitor employing its chemotherapeutic activity via the inhibition of RAF/MEK/ERK pathway, platelet-derived growth factor receptor beta (PDGFR-β), tyrosine kinases and vascular endothelial growth factor receptor (VEGFR)^12,33^. The promising responses attained by sorafenib, which resulted in a 9.2-month median overall survival, encouraged the FDA in 2016 to announce sorafenib for the treatment of advanced HCC^34^. However, the lack of improvement in patients’ quality of life, symptoms caused by the disease progression due to the need of large doses of sorafenib^35^ and the emergence of resistance^36,37^ diminished the beneficial effects of sorafenib in the management of HCC patients.

To enhance the anticancer activity of sorafenib, the combination of sorafenib with other chemical drugs was assessed in several studies. For example, sorafenib combined with doxorubicin was performed in a double-blind phase II study, resulting in prolonged progression-free survival and overall survival compared with doxorubicin monotherapy^38^. In addition, a previous study had shown that the combination of sorafenib with a lipid-lowering drug, fluvastatin significantly reduced HCC tumor development *in vivo*^39^. Moreover, many studies have indicated the synergy of sorafenib with different bioactive components in plant extracts such as corosolic acid in *Actinidia chinensis*^16^, resveratrol in grapes, peanuts and red wine^17^ and wogonin (5,7-dihydroxy-8-methoxyflavone) in *Scutellaria baicalensis*^18^.

While the hepatoprotective effects of *A*. *cinnamomea* have been reported by many studies, reports about the antitumor activity of *A*. *cinnamomea* against liver cancer are few^40^. The current study showed the synergistic combination of sorafenib with EAC on decreasing cell survival of HepG2 and Huh-7 cells. We found that EAC sensitizes HCC cells towards sorafenib-induced apoptosis as demonstrated by cellular and nuclear morphological changes, Annexin-V staining and caspase 3 activation. The ability of *A*. *cinnamomea* fruiting bodies to induce apoptosis in liver cancer cells was reported previously^41^. Low doses of EAC and sorafenib that showed synergistic effects were selected for further molecular studies *in vitro*. Our results showed that treatment of HCC cells with EAC decreased ERK phosphorylation dose-dependently and boosted the inhibitory effect of sorafenib ERK phosphorylation. The same synergistic effect on ERK phosphorylation was observed *in vivo*, suggesting that the EAC/sorafenib combination synergy is, in part, mediated through inhibition of ERK phosphorylation *in vitro* and *in vivo*. This finding is remarkable since the inhibition of ERK phosphorylation is the major anticancer mechanism of sorafenib and p-ERK is a hallmark indicator of HCC sensitivity to sorafenib^12,42^.

In MTT assay, HepG2 cells were more sensitive to sorafenib and EAC as single-agents than Huh-7 cells. However, during combination treatment, sorafenib/EAC combination showed a greater effect on Huh-7 cells. This phenomenon may be caused by their genetic characteristics. Many studies indicated that p53 gene expression is involved in the regulation of cell apoptosis during chemotherapeutic drugs treatment. Furthermore, analysis of p53 in Huh-7 and HepG2 cell lines have shown that HepG2 cells carry wild-type p53, whereas Huh7 cells have point mutations at p53 codon 220^43,44^. We speculate that this could attribute to their different responses to the sorafenib/EAC combination.

The effect of sorafenib/EAC combination on HCC cell cycle progression caused a significant arrest in the G2/M phase, which was due to the downregulation of cyclin proteins. This finding was in line with the reported ability of both sorafenib and *A*. *cinnamomea* to induce cell cycle arrest in cancer cells^45,46^. In addition, the combination inhibited the invasiveness of HCC as indicated by the ability to prevent cancer cell migration in the scratch assay. This inhibitory activity was parallel to the transcriptional inhibition of matrix metalloproteinases, MMP2 and MMP9. It was reported that the protective effect of EAC against ethanol-induced liver injury was mediated through the suppression of MMP-9^47^. These results collectively showed the ability of EAC to counteract cancer cell resistance to sorafenib, which could be attributed to EAC inhibitory effects on cell survival pathways.

For the *in vivo* study, sorafenib/EAC combination at the same doses that showed a minimal effect when used as a single-agent, caused a significant tumor shrinkage when given as a combination. The *in vivo* antitumor activity of the combination was accompanied by a decrease in p-ERK, which was consistent with the observed mechanism *in vitro*. In addition, sorafenib/EAC combination was safe in mice and did not show any signs of avert toxicity in liver, kidney or spleen of the mice. It is worthy to mention that we observed an increase in the size of the xenograft tumor upon single treatment with 100 mg/kg EAC. This could be attributed to the small dose of EAC used compared to a previous study which indicated that EAC from *A*. *cinnamomea* fruiting body can reduce tumor development with a treatment dose of 300 mg/kg in xenograft tumor model^48^.

In conclusion, the current study provided valuable information for liver cancer patients who are undergoing sorafenib therapy. The results highlighted evidence that EAC can boost the inhibitory effect of sorafenib on ERK phosphorylation in HCC cells. However, the translational potential of sorafenib/EAC combination into the clinical application requires more *in vivo* investigation including multiple daily dosing and pharmacokinetics. Finally, the safety and significant inhibitory effect of sorafenib/EAC combination on cancer cell suggest that the use of EAC could be further explored as a potential adjuvant to reinforce the application of sorafenib therapy in HCC.

## Methods

### Reagents

Fetal bovine serum (FBS), MEM medium, penicillin, streptomycin, and trypsin-EDTA were obtained from GIBCO BRL (Gaithersburg, MD USA). Dimethyl sulfoxide (DMSO), methanol, ethanol, acetic acid, acrylamide/bis TEMED (tetramethylenediamine), 3-(4,5-dimethylthiazol-2-yl)-2,5-diphenyl-tertazolium bromide (MTT), were purchased from Sigma-Aldrich (St. Louis, MO, USA). Antibodies against Bcl-2, cdc2, cdc25, NF-κB and p-NF-κB were purchased from Santa Cruz Biotechnology (Dallas, TX USA). While the antibodies against cyclin A, cyclinB1, JNK, p-JNK (pT183/pY185), p38, p-p38 (pT180/pY182), ERK, p-ERK (pT202/pY204), Ki67 and β-actin were obtained from Cell Signaling Technology (Danvers, MA. USA). Secondary antibodies against mouse and rabbit antibodies were from Jackson ImmunoResearch (West Grove, PA, USA). *A*. *cinnamomea* strain (BCRC 35396) was obtained from Bioresource Collection and Research Center (Hsinchu, Taiwan).

### Cell lines and animal

Hepatocellular carcinoma cells (HepG2 and Huh-7) were purchased from ATCC (Rockville, MD, USA). The cells were grown in Dulbecco’s Modified Eagle Medium (DMEM) supplemented with 10% fetal bovine serum (FBS). BALB⁄c and NOD-SCID mice (18–20 gm and 6–8 weeks old) were purchased from Laboratory Animal Center of National Cheng Kung University. All animal handling and procedure were according to and approved by the Institutional Animal Care and Use Committee of Chia Nan University of Pharmacy and Science (CN-IACUC NO. 104026).

### Preparation of *A*. *cinnamomea* extract

*A*. *cinnamomea* was incubated in M25 medium (2% Glucose, 0.1% peptone, 2% Malt extract and 2% agar) at 25 °C for 50 days. After incubation, the plates were dried in a freeze dryer and extracted by 95% and 75% ethanol every 3 days. The total crude extracts were concentrated using a rotary evaporator. The dry extracts were dissolved in DMSO.

### Identification of the major components of the extracts by LC/MS analysis

Filtration through 0.45 µm membrane filter was performed for all samples before the injection into LC. 20 µL of 30 µg/ml of the extracts were injected individually into a Hitachi LC system (Hitachi, Tokyo, Japan). The major components of the extracts were identified essentially as detailed before^49^.

### Cell viability assay

MTT assay was employed to measure the cell viability as mentioned before^50^. DMSO was used as a vehicle for all extracts and sorafenib, where the final concentration of DMSO doesn’t exceed 0.1% (v/v) in the culture media. Cells were treated with vehicle, sorafenib (0.5, 1, 2.5, 5, 7.5, 10, 25 µM) or EAC (10, 25, 50, 100, 150, 200, 250 µg/ml) for 48 h. Media were then replaced with 0.5 mg/mL MTT in fresh media and incubated for another 4 h. The formed formazan crystals were then solubilized in DMSO and the optical density was measured at 570 nm.

### Cell cycle analysis

Cells were treated with sorafenib with or without various concentrations of EAC for 24 and 48 h and cell cycle was analyzed as mentioned before^51^.

### Assessment of cellular and nuclear morphology

The changes of nuclear chromatin morphology in the treated cells experiencing apoptosis were identified by staining with DAPI after fixation with 3% paraformaldehyde. Fixed stained cells were imaged using fluorescence microscopy.

### Immunoblotting

Cells or tumor tissues were harvested, and total cell lysates were extracted, separated on SDS-polyacrylamide gels and transferred onto PVDF or nitrocellulose membranes as described before^52^. The membranes were probed with p-p38, p38, p-ERK, ERK, p-JNK, JNK, cyclin A, cyclin B1, cdc2, cdc 25, Bcl-2, NF-κB (p65), p-NF-κB (pp65) and β-actin primary antibodies. Then, corresponding, anti-mouse IgG or anti-rabbit IgG secondary antibodies, linked to horseradish peroxidase were used. The blots were visualized by ECL™ WB Detection Reagent (Millipore, Billerica, MA, USA) per manufacturer’s instructions.

### Annexin V/propidium iodide apoptosis assay

The assessment of the ability of different extracts to trigger programmed cell death in HCC cells was performed using Annexin V/Propidium Iodide flow cytometry. Briefly, 2 × 10^6^ cells/well were seeded in 6-well plates for 24 h. Cells were then treated with vehicle, 2.5 µM sorafenib with or without 100 µg/ml EAC for 48 h. Flow cytometric analysis was performed as mentioned before^51^.

### Wound healing assay

The assay was performed as detailed before^53^. The monolayer culture of seeded HCC cells was scraped with a sterile micropipette tip to form a gap of a constant width. Cells were then treated with vehicle, 2.5 µg/ml sorafenib with or without 100 µg/ml EAC for 36 h. The migration of cells into wounded areas was evaluated with an inverted microscope.

### Zymography assay

For zymography assay, Huh-7 cells were seeded at 1.2 × 10^6^ per well in 6-well flat-bottomed plates and incubated in 10% FBS-supplemented DMEM for 16 h. Media was removed and cells were washed with PBS for three times. Cells were then treated with 2.5 μM sorafenib, 100 μg/ml EAC or 2.5 μM sorafenib/100 μg/ml EAC in FBS-free media for 16 h. The FBS-free cell culture medium was collected and concentrated by protein concentrator (Millipore, Darmstadt, Germany). The activity of MMP-2 and MMP-9 was determined by 10% acrylamide gel containing 0.1% gelatin. After running, the gel was washed twice with washing buffer (2.5% triton X-100, 50 mM Tris-HCl/pH 7.5, 5 mM CaCl_2_ and 1 μM ZnCl_2_) for 30 min, and the gel was incubated with incubation buffer (1% triton X-100, 50 mM Tris-HCl/pH 7.5, 5 mM CaCl_2_ and 1 μM ZnCl_2_) for 24 h at 37 °C. Areas of MMP-2 and MMP-9 enzyme activity appear as white bands after staining with Coomassie blue dye. The ratio of white bands on the gel was evaluated using ImageJ software.

### Boyden chamber invasion assay

Boyden chamber invasion assay was performed as detailed before^54^. First, the polycarbonate filter (8 μm pore) was pre-coated with Matrigel. 6 × 10^5^ cells were collected after sorafenib, EAC or sorafenib/EAC treatment, and added to the upper chamber in serum-free medium. The lower chamber was filled with FBS medium as a chemoattractant, and the chamber was incubated in CO_2_ incubator for 24 h at 37 °C. The invaded cells on the lower surface of the membrane were fixed by methanol and analyzed by Giemsa staining assay. The number of invaded cells on the filter membrane from five individual fields under microscopy (200× magnification) were scored.

### Analysis of caspase-3 activity

HCC cells were treated with sorafenib (2.5 µM) with or without EAC (100 µM) for 48 h and caspase-3 activity was assessed using BD Pharmingen™-Caspase-3, Active Form, Apoptosis Kit according to manufacturer’s instructions.

### Xenograft model of HCC and histopathological examination

Before establishing the xenograft model, the safety of the test compounds and the proposed combinations were tested in naive BALB/c mice. Animals (*n* = 3/group) were randomly assigned to 4 groups. Mice received i.p. (intraperitoneal) injection of vehicle (PBS), sorafenib (25 mg/kg), EAC (100 mg/kg) or sorafenib (25 mg/kg)/EAC (100 mg/kg) every other day for 7 weeks. The dose of sorafenib and EAC as single agents were selected to be similar to that used in the combination for a fair comparison and to test the synergy in the combination. The animals were then sacrificed, and liver, kidney and spleen were collected and analyzed. HCC xenografts were established in NOD-SCID mice by subcutaneous inoculation of about 5 × 10^6^ Huh-7 cells suspension in phosphate-buffered saline in the flank region. After one week of inoculation, when the tumors were palpable, mice (*n* = 7/group) were assigned randomly to 4 groups. Sorafenib and EAC stock solution were dissolved in PBS to make a total volume of 100 μl of 2.5 mg/kg sorafenib, 100 mg/kg EAC or 2.5 mg/kg sorafenib/100 mg/kg EAC. The animals received i.p. injection every other day of vehicle (normal saline), 2.5 mg/kg sorafenib, 100 mg/kg EAC or 2.5 mg/kg sorafenib/100 mg/kg EAC combination for 7 weeks. Similar treatment protocols for sorafenib combination with other agents were reported before^55,56^. The tumor volume was assessed by the formula of a rational ellipsoid: [Volume = (shorter axis) *m*_1_^2^ × (longer axis) *m*_2_ × 0.5236], and the tumor volume was measured once a week. At the end of treatment, when the tumor diameter exceeded 20 mm in some animals at week 7 (Supplementary Material Part 3), mice were sacrificed, and the tumors were collected then subjected to histopathological and immunohistochemical examination as detailed before^2^.

### Statistical analysis

Data were presented as mean ± standard deviations. The multiple group comparisons were done via one-way analysis of variance (ANOVA) and Tukey-Kramer test was utilized as a post-hoc test. The differences were considered significant at *P < 0.05, **P < 0.01 and ***P < 0.001. The GraphPad InStat software, version 3.05 was used for statistical analysis. Graphs were plotted using GraphPad Prism software, version 5.00 (GraphPad Software, Inc. La Jolla, CA).

## Electronic supplementary material

Supplementary material

## Data Availability

All data generated or analyzed during this study are included in this article and its Supplementary Information files.
