# Supplementary material for: Antrodia cinnamomea boosts the anti-tumor activity of sorafenib in xenograft models of human hepatocellular carcinoma
Source: Sci Rep. 2018 Aug 27;8:12914. doi: 10.1038/s41598-018-31209-8 (PMC6110745; doi:10.1038/s41598-018-31209-8)
Supplement: Supplementary file 1 — Supplementary material [file 41598_2018_31209_MOESM1_ESM.docx]

**Supplementary Material**

***Antrodia cinnamomea* boosts the anti-tumor activity of sorafenib in xenograft models of human hepatocellular carcinoma**

### Wei-De Wu^1,#^, Pin-Shern Chen^1,#^, Hany A. Omar^2,4^, El-Shaimaa A. Arafa^3,4^, Hung-Wei Pan^5^, JingYueh Jeng^1^, Jui-Hsiang Hung^1,6*^

^1^Department of Biotechnology, Chia Nan University of Pharmacy and Science, Tainan, Taiwan

^2^Sharjah Institute for Medical Research and College of Pharmacy, University of Sharjah, Sharjah, UAE

^3^Department of Pharmacology, College of Pharmacy, Ajman University, Ajman, UAE.

^4^Department of Pharmacology, Faculty of Pharmacy, Beni-Suef University, Beni-Suef, Egypt

^5^Department of Medical Education and Research, Kaohsiung Veterans General Hospital, Kaohsiung, Taiwan.

^6^Drug Discovery and Development Center, Chia Nan University of Pharmacy and Science, Tainan, Taiwan

# These authors contributed equally to this work.

*** Corresponding author:** Department of Biotechnology, Chia Nan University of Pharmacy and Science, Tainan, Taiwan. Tel.: +886 6 2664911-2549; fax: +886 6 2662135.

*E-Mail address*: [hung86@mail.cnu.edu.tw](mailto:hung86@mail.cnu.edu.tw)

***Part 1: The metabolite profiles of EACF and EAC as determined by HPLC/LC-MS/MS***

**EACF profiles:**

**mAU**

**LC/UV, 270 nm**

2 / 5 / 6

4

3

8

7

1

**Minutes**

EIC 482+AIMS

EIC 468+AIMS

EIC 526+AIMS

EIC 470+AIMS

EIC 484+AIMS

EIC 486+AIMS

EIC 488+AIMS

EIC 454+AIMS

Compound 1

Compound 2

Compound 3

Compound 4

Compound 5

Compound 6

Compound 7

Compound 8

**Minutes**

**EAC profiles:**

**LC/UV, 270 nm**

**mAU**

2 / 5 / 6

4

3

8

7

1

**Minutes**

**Minutes**

EIC 482+AIMS

EIC 468+AIMS

EIC 526+AIMS

EIC 470+AIMS

EIC 484+AIMS

EIC 486+AIMS

EIC 488+AIMS

EIC 454+AIMS

Compound 1

Compound 2

Compound 3

Compound 4

Compound 5

Compound 6

Compound 7

Compound 8

***Part 2: Full Length gels***

**Figure 3H**

Cyclin A


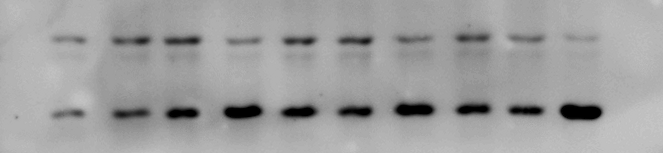


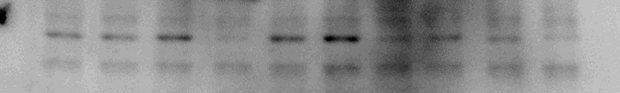
Cyclin B1


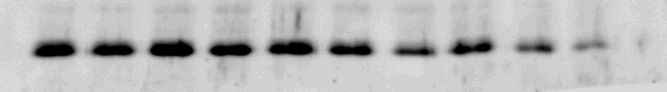
Cdc2

Cdc25


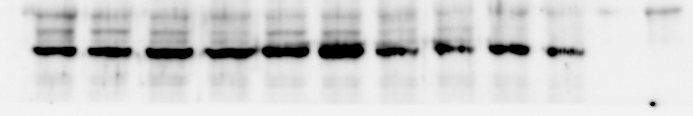


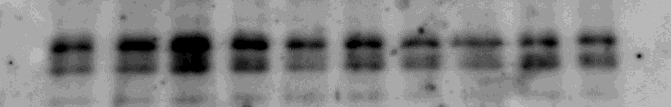
Bcl-2

β-actin


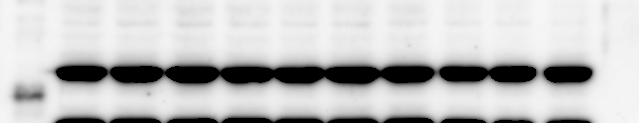


**Figure 4**

p-ERK


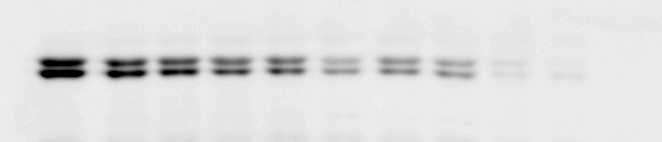


ERK


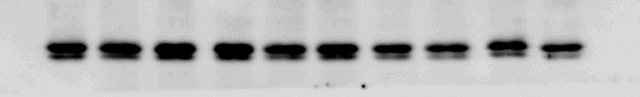


p-p38


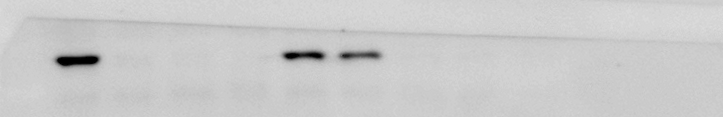


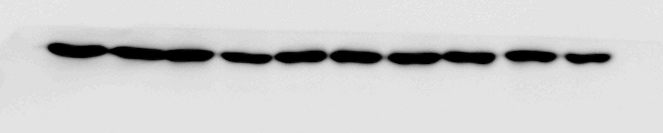
P38


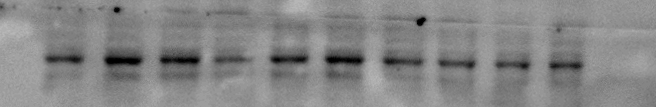
p-JNK


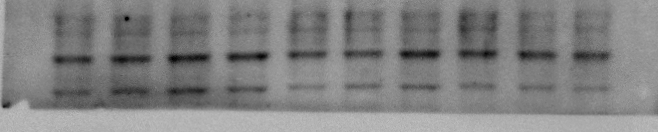
JNK


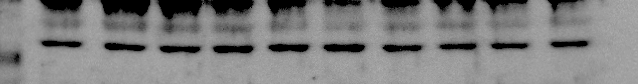
β-actin

**Figure 6E**

+

+

+

-

-

+

-

-

EAC

-

-

+

-

-

+

+

+

Sorafenib


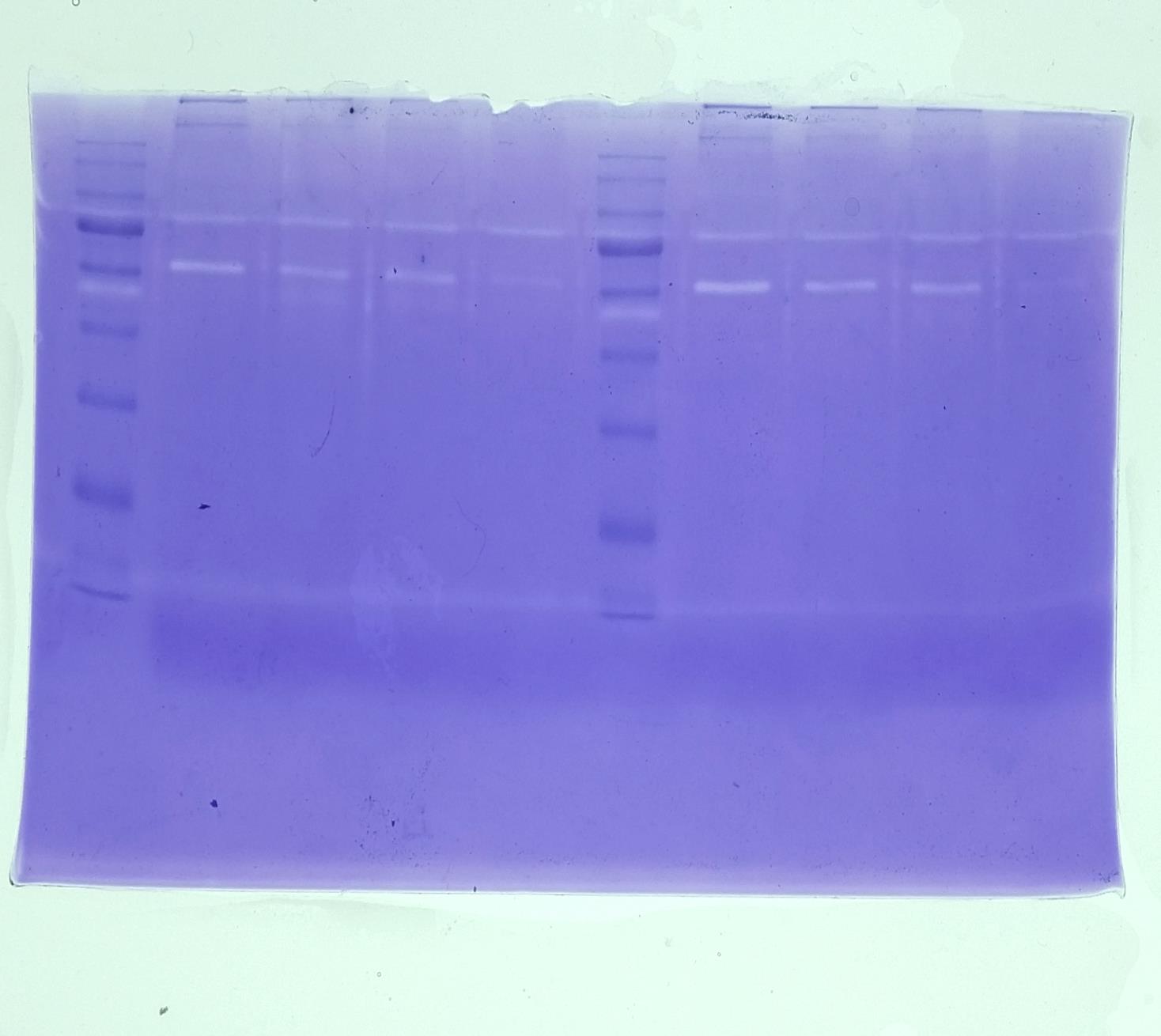


**Figure 7C**

p-ERK


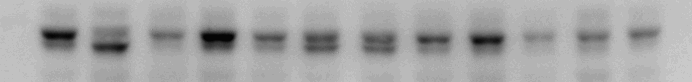


ERK


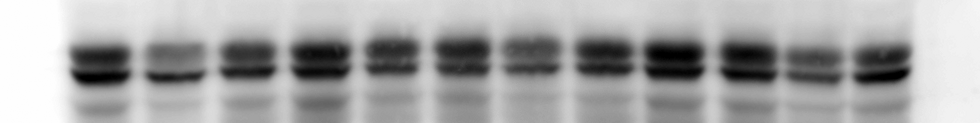


β-actin
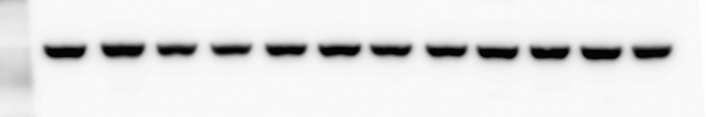


***Part 3: Xenograft model of HCC***

1. Cell number: 5x10^6^ cells/ml
2. The experimental group: control (PBS), sorafenib (2.5 mg/kg), EAC (100 mg/kg) or sorafenib (2.5 mg/kg)/EAC (100 mg/kg)
3. Tumor size: The formula is Volume = (Length × Width^2^) **×** 0.52; mm^3^

**Raw data of control group (Length / Width); mm**

|  | **#1** | **#2** | **#3** | **#4** | **#5** |
| --- | --- | --- | --- | --- | --- |
| **Week 1** | 2 / 1 | 3 / 2 | 2 / 2 | 3 / 2 | 2 / 1 |
| **Week 2** | 4 / 4 | 8 / 1 | 5 / 2 | 10 / 4 | 5 / 2 |
| **Week 3** | 6 / 4 | 9 / 7 | 10 / 7 | 8 / 6 | 8 / 5 |
| **Week 4** | 14 / 12 | 11 / 9 | 13 / 12 | 14 / 9 | 9 / 9 |
| **Week 5** | 15 / 14 | 16 / 16 | 14 / 12 | 14 / 14 | 13 / 13 |
| **Week 6** | 16 / 15 | 16 / 14 | 18 / 17 | 18 / 15 | 18 / 15 |
| **Week 7** | 19 / 17 | 20 / 20 | 20 / 20 | 18 / 17 | 19 / 18 |

**Raw data of sorafenib group (Length / Width); mm**

|  | **#1** | **#2** | **#3** | **#4** | **#5** |
| --- | --- | --- | --- | --- | --- |
| **Week 1** | 2 / 2 | 4 / 2 | 2 / 1 | 3 / 1 | 2 / 1 |
| **Week 2** | 6 / 2 | 6 / 3 | 3 / 3 | 6 / 4 | 5 / 2 |
| **Week 3** | 5 / 3 | 7 / 6 | 9 / 8 | 6 / 6 | 10 / 8 |
| **Week 4** | 12 / 10 | 11 / 10 | 6 / 5 | 11 / 8 | 14 / 10 |
| **Week 5** | 8 / 6 | 16 / 14 | 14 / 11 | 12 / 11 | 16 / 12 |
| **Week 6** | 11 / 9 | 15 / 11 | 18 / 18 | 20 / 13 | 16 / 16 |
| **Week 7** | 15 / 14 | 19 / 18 | 20 / 18 | 20 / 20 | 20 / 19 |

**Raw data of EAC group (Length / Width); mm**

|  | **#1** | **#2** | **#3** | **#4** | **#5** |
| --- | --- | --- | --- | --- | --- |
| **Week 1** | 4 / 2 | 2 / 1 | 3 / 1 | 2 / 2 | 2 / 2 |
| **Week 2** | 9 / 5 | 6 / 4 | 8 / 5 | 7 / 6 | 5 / 3 |
| **Week 3** | 8 / 6 | 10 / 7 | 7 / 6 | 14 / 9 | 9 / 8 |
| **Week 4** | 14 / 12 | 18 / 12 | 12 / 12 | 10 / 10 | died |
| **Week 5** | 14 / 14 | 18 / 15 | 15 / 13 | 18 / 13 | died |
| **Week 6** | 20 / 14 | 20 / 19 | 17 / 15 | 16 / 15 | died |
| **Week 7** | 20 / 20 | 20 / 19 | 20 / 20 | died | died |

**Raw data of sorafenib/EAC group (Length / Width); mm**

|  | **#1** | **#2** | **#3** | **#4** | **#5** |
| --- | --- | --- | --- | --- | --- |
| **Week 1** | 3 / 2 | 2 / 2 | 2 / 2 | 1 / 1 | 2 / 1 |
| **Week 2** | 4 / 2 | 3 / 1 | 4 / 2 | 2 / 1 | 4 / 2 |
| **Week 3** | 2 / 1 | 8 / 4 | 7 / 3 | 7 / 4 | 6 / 4 |
| **Week 4** | 10 / 6 | 10 / 6 | 12 / 6 | 10 / 6 | 5 / 3 |
| **Week 5** | 8 / 7 | 12 / 10 | 12 / 9 | 13 / 10 | 12 / 10 |
| **Week 6** | 14 / 10 | 14 / 11 | 13 / 8 | 10 / 7 | 12 / 11 |
| **Week 7** | 16 / 14 | 15 / 13 | 12 / 10 | 15 / 13 | 16 / 13 |

1. Tumor volume

**Control group****: (Lenght×Width^2^)** **× 0.52; mm^3^**

|  | **#1** | **#2** | **#3** | **#4** | **#5** |
| --- | --- | --- | --- | --- | --- |
| **Week 1** | 1.04 | 6.24 | 4.16 | 6.24 | 1.04 |
| **Week 2** | 33.28 | 4.16 | 10.4 | 83.2 | 10.4 |
| **Week 3** | 49.92 | 229.32 | 254.8 | 149.76 | 104 |
| **Week 4** | 1048.32 | 463.32 | 973.44 | 589.68 | 379.08 |
| **Week 5** | 1528.8 | 2129.92 | 1048.32 | 1426.88 | 1142.44 |
| **Week 6** | 1872 | 1630.72 | 2705.04 | 2106 | 2106 |
| **Week 7** | 2855.32 | 4160 | 4160 | 2705.04 | 3201.12 |

**Sorafenib group: (Lenght×Width^2^) × 0.52; mm^3^**

|  | **#1** | **#2** | **#3** | **#4** | **#5** |
| --- | --- | --- | --- | --- | --- |
| **Week 1** | 4.16 | 8.32 | 1.04 | 1.56 | 1.04 |
| **Week 2** | 12.48 | 28.08 | 14.04 | 49.92 | 10.4 |
| **Week 3** | 23.4 | 131.04 | 299.52 | 112.32 | 332.8 |
| **Week 4** | 624 | 572 | 78 | 366.08 | 728 |
| **Week 5** | 149.76 | 1630.72 | 880.88 | 755.04 | 1198.08 |
| **Week 6** | 463.32 | 943.8 | 3032.64 | 1757.6 | 2129.92 |
| **Week 7** | 1528.8 | 3201.12 | 3369.6 | 4160 | 3754.4 |

**EAC group: (Lenght×Width^2^) × 0.52; mm^3^**

|  | **#1** | **#2** | **#3** | **#4** | **#5** |
| --- | --- | --- | --- | --- | --- |
| **Week 1** | 8.32 | 1.04 | 1.56 | 4.16 | 4.16 |
| **Week 2** | 117 | 49.92 | 104 | 131.04 | 23.4 |
| **Week 3** | 149.76 | 254.8 | 131.04 | 589.68 | 299.52 |
| **Week 4** | 1048.32 | 1347.84 | 898.56 | 520 | died |
| **Week 5** | 1426.88 | 2106 | 1318.2 | 1581.84 | died |
| **Week 6** | 2038.4 | 3754.4 | 1989 | 1872 | died |
| **Week 7** | 4160 | 3754.4 | 4160 | died | died |

**Sorafenib/EAC group: (Lenght×Width^2^) × 0.52; mm^3^**

|  | **#1** | **#2** | **#3** | **#4** | **#5** |
| --- | --- | --- | --- | --- | --- |
| **Week 1** | 6.24 | 4.16 | 4.16 | 0.52 | 1.04 |
| **Week 2** | 8.32 | 1.56 | 8.32 | 1.04 | 8.32 |
| **Week 3** | 1.04 | 66.56 | 32.76 | 58.24 | 49.92 |
| **Week 4** | 187.2 | 187.2 | 224.64 | 187.2 | 23.4 |
| **Week 5** | 203.84 | 624 | 505.44 | 676 | 624 |
| **Week 6** | 728 | 880.88 | 432.64 | 254.8 | 755.04 |
| **Week 7** | 1630.72 | 1318.2 | 624 | 1318.2 | 1406.08 |
